# Assessment of the Composition of Breastmilk Substitutes, Commercial Complementary Foods, and Commercial Snack Products Commonly Fed to Infant and Young Children in Lebanon: A Call to Action

**DOI:** 10.3390/nu15051200

**Published:** 2023-02-27

**Authors:** Maha Hoteit, Carla Ibrahim, Joanna Nohra, Yonna Sacre, Lara Hanna-Wakim, Ayoub Al-Jawaldeh

**Affiliations:** 1 Food Sciences Unit, National Council For Scientific Research (CNRS), Beirut P.O. Box 11-8281, Lebanon; 2 PHENOL Research Group (Public HEalth Nutrition prOgram Lebanon), Faculty of Public Health, Lebanese University, Beirut 6573, Lebanon; 3 Lebanese University Nutrition Surveillance Center (LUNSC), Lebanese Food Drugs and Chemical Administrations, Lebanese University, Beirut 6573, Lebanon; 4 Doctoral School of Sciences and Technology (DSST), Lebanese University, Hadath 1533, Lebanon; 5 Department of Nutrition and Food Sciences, Faculty of Arts and Sciences, Holy Spirit University of Kaslik (USEK), Jounieh P.O. Box 446, Lebanon; 6 Department of Agricultural and Food Engineering, School of Engineering, Holy Spirit University of Kaslik (USEK), Jounieh P.O. Box 446, Lebanon; 7 World Health Organization Regional Office for the Eastern Mediterranean, Cairo 11371, Egypt

**Keywords:** infant formula, baby food product, nutrient composition, Lebanon

## Abstract

(1) Background: Nutrition for optimum growth and physical development is acquired by adequate infant feeding practices. (2) Methods: One hundred seventeen different brands of infant formulas (n = 41) and baby food products (n = 76) were selected from the Lebanese market and were analyzed for their nutritional content. (3) Results: Saturated fatty acid content was detected to be the highest in follow-up formulas (79.85 g/100 g) and milky cereals (75.38 g/100 g). Among all saturated fatty acids, palmitic acid (C16:0) accounted for the greatest proportion. Moreover, glucose and sucrose were the predominant added sugars in infant formulas, while sucrose was the main added sugar in baby food products. Our data showed that the majority of the products were non-compliant to the regulations and the manufacturers’ nutrition facts labels. Our results stated also that the contribution to the daily value for the saturated fatty acids, added sugars, and protein exceeded the daily recommended intake for most infant formulas and baby food products. (4) Conclusions: This requires careful evaluation from policymakers in order to improve the infant and young children feeding practices.

## 1. Introduction

Nutrition for optimum growth and physical development is acquired by adequate infant feeding practices. It has been proven that a child’s first 1000 days are vital to their development [1]. Throughout this time, children are growing more rapidly than any other point in their lives [1]. According to UNICEF estimates, 149 million children under the age of five have stunted growth and development as a result of a chronic shortage of nutrient-rich food in their diets, and 45 million children under the age of five experience wasted growth [2]. Therefore, an inadequate nutritional status can lead to serious health problems in infants and young children [3].

Breastmilk is the golden standard for a child’s health and survival [4]. It is a complete food rich in nutrients such as carbohydrates, protein, fat, vitamins, minerals, digestive enzymes, and hormones [5]. Moreover, breastmilk offers a variety of bioactive substances, such as oligosaccharides, that support the growth of a robust immune system and a healthy microbiota [6]. However, each woman’s breastmilk is distinct and depends on a variety of elements, such as her diet, general health, and the infant’s needs [7]. That being said, manufacturers aim at producing infant formulas that closely as possible resemble human breastmilk’s nutritional profile [8], so that most babies who receive alternative forms of feeding, when breastfeeding may not be appropriate or feasible, to meet all or part of their nutritional needs [5]. The International Code of Marketing of Breastmilk Substitutes (the Code) was published by the World Health Assembly in 1981 and written in response to the marketing activities of the infant feeding industry which were promoting formula feeding over breastfeeding, and in turn leading to dramatic increases in maternal and infant morbidity and mortality [9]. Despite all the regulations that have been put in place, irresponsible marketing of breastmilk substitutes hinders global efforts to increase the rate and duration of breastfeeding [4]. Moreover, poor implementation of the labels’ nutritional facts, packaging design features, and health claims are of utmost concern [7]. Many of the standards were substantially affected by infant formula companies, who put profits before the health of children [10].

According to the WHO guidelines, infants should be exclusively breastfed for the first six months of their lives, and thereafter consume nutrient-adequate and appropriate complementary foods with continued breastfeeding to two years of age or beyond [11]. Complementary feeding is a crucial phase and depends on many factors; including food availability and access, local culture, and pediatrician’s guidance; therefore, any failure can result in a long-term consequence that can last into adulthood [12,13,14]. In addition, the baby and toddler food market has grown over the years and its applications have expanded in tandem with the rise in working mothers [15]. Given that dietary habits can form as early as 2–3 years of age and last a lifetime, the wide range of products that are readily available can perplex family decisions [15].

Hence, our study is the first national study that evaluated the composition of infant formulas and baby food products, thus we intended (1) to assess the energy, fat, carbohydrate, sugar, protein, ash, moisture, and chloride contents of infant formulas and baby food products available in the Lebanese market, (2) to determine the level of compliance of infant formulas and baby food products with Codex, EFSA, and Libnor standards, (3) to tackle the difference between the infant foods actual nutrient content and their label claims, and (4) to evaluate the adequacy of the nutritional composition of these products with the daily recommended value in infants.

## 2. Materials and Methods

### 2.1. Data Collection

A total of 117 different brands of infant formulas (n = 41): 13 starting formulas (0–6 months), 3 follow-up formulas (6–12 months), 7 growing-up formulas (1–3 years), and 18 extra-care formulas (0–12 years), and commercial baby food products (n = 76): 16 cereals (5 milky cereals and 11 cereal meals), 21 cornflakes, 7 biscuits, and 32 pureed foods (12 fruit puree, 6 vegetable and legume puree, 12 meat or fish puree with vegetables, and 2 milk-based puree) were selected, based on their availability and high purchasing levels from pharmacies and supermarkets in Lebanon. Infant and toddler food products were classified according to their primary ingredient showing on the jars of the pureed foods and the carton back of the cereals, biscuits, and cornflakes. Additionally, all complementary foods indicated the corresponding age of use, which was 6 months and above. Information on the containers of each formula and baby food products was carefully examined and translated in an excel spreadsheet; all data were collected from the nutrition information panel (NIP) on the back of the package and information regarding the infant formula and baby food products was adjusted to meet the same measuring unit of the analyzed products. Prior to the analysis, all samples were coded and retained in an appropriate humidity and temperature conditions.

### 2.2. Chemical Analysis of Infant Formulas and Baby Food Products

#### 2.2.1. Fat Content Analysis

Fat was extracted using the Rose Gotlieb method. In order to disassemble the bonds that hold the lipid and non-lipid components together, the sample was treated with hydrochloric acid and ethyl alcohol. Organic solvents (di-ethyl ether and Hexane) were then added to extract the fat. The solvent was evaporated, and the residue was weighted.

#### 2.2.2. Fatty Acid Profile Analysis

A total of 0.5 g of extracted fat was boiled for 5 min with 5 mL of 0.5 N methanolic KOH to separate the fatty acids from the glycerol. Esterification of fatty acids was performed by refluxing by boiling for 15 min with 15 mL of an esterification reagent. The fatty acid methyl esters were then extracted in a separatory funnel with 25 mL of diethyl ether. The organic layer was washed twice with 25 mL distilled water and diluted with diethyl ether after the aqueous layer was discarded. The fatty acids were identified by comparing them to a chromatogram of commercially available fatty acid methyl esters, and their area percentages were calculated. Fatty acid results were expressed as %wt/wt of fatty acids.

#### 2.2.3. Protein Content Analysis

According to AOAC, the formal titration method was used to determine the protein concentration. The protein content of infant formulas and baby foods was obtained by multiplying the nitrogen content 6.38 dairy product factor (N × 6.38).

#### 2.2.4. Carbohydrate Content Analysis

The total of the protein, fat, moisture, and ash values was deducted from 100 to calculate the total carbohydrate content of infant formulas and baby foods.

#### 2.2.5. Total Sugars

The Munson and Walker principle was used to calculate total sugars. Fehling’s solution was boiled with a solution containing reducing sugars, and converted to insoluble dark red cuprous oxide (Cu_2_O). The precipitate obtained was filtered using a special perforated crucible and then weighed. Reducing or total sugars were then calculated from tables depending on the weight of cuprous oxide precipitate.

#### 2.2.6. Total Energy Analysis

To calculate the total energy, Atwater coefficients for protein, carbohydrate, and fat were used. For each gram of protein, carbohydrate, and fat, the standard values of 4, 4, and 9 calories per gram were applied, respectively. The following equation was used to determine calories:

Caloric content per 100 g or mL = (4 × protein) + (4 × carb) + (9 × fat)

#### 2.2.7. Ash Content Analysis

The ash content is determined by calculating the weight loss caused by drying the sample in a high temperature muffle furnace (500–600 °C), which causes water and other volatile materials to vaporize and organic substances to be converted to CO_2_, H_2_O, and N_2_ in the presence of oxygen in the air.

#### 2.2.8. Moisture Content Analysis

A 3 g sample was dried in a convection drying oven at 105 ± 3 °C for 3 h. The sample was then covered, placed in a desiccator to cool, and weighed when it reached room temperature. The procedure was repeated until the weight was constant.

#### 2.2.9. Chloride Analysis

Titration was used to determine the concentration of chloride. Chloride was extracted from the sample (except for water) by ashing at 550 °C. After dissolving the ash in distilled water, the chlorides were titrated with silver nitrate, resulting in a silver chloride precipitate. The titration was finished when all of the chloride ions precipitated.

### 2.3. Statistical Analysis

All data were analyzed using IBM SPSS Statistics for Windows, Version 22.0. IBM Corp, Armonk, NY, USA. The data obtained were presented as the mean and standard deviations (SD). The paired sample *t* test was used to determine the compliance of the measured values of the infant formulas and baby food products with the labeling at *p* < 0.05. The percentage of fatty acids of the labeled products was converted to % of total fatty acids and compared to the measured values.

In order to evaluate our results with the regulations, the total grams or milligrams per 100 kcal was calculated for infant formulas and baby food products by dividing the measured value (g or mg/100 g) by the total calories (per 100 g) and then multiplying the result by 100.

As for the percentage of daily value (DV), it was calculated using the following equation:

$$\%\mathrm{DV}:\;\frac{\mathrm{Measured}\;\mathrm{value}*}{\mathrm{daily}\;\mathrm{value}}\times 100$$

The US Food and Drug Administration (FDA) defines the daily value (DV) as “reference values for reporting nutrients on the nutrition labels”. Furthermore, %DV helps the consumer determine whether the serving of food and its nutrient content is high (>20%), good (10–19%), or low (10%) [16].

* The measured value was converted from g/100 g to g/100 mL and then used to calculate the percentage of daily value:

Each 30 mL of milk needs 1 scoop of powdered milk, equal to 4 g. This information was retrieved from the recommendation mentioned on the back of the package.
Step 1: 100 mL of milk = $\frac{100\;\left(\mathrm{mL}\right)\;\times \;4\left(g\right)}{30\;\left(\mathrm{mL}\right)}$ = 13.3 g
Step 2: Measured value in g/100 mL = $\frac{13.3\;\left(g\right)\;\times \;\mathrm{measured}\;\mathrm{value}\;\left(g\right)}{100\;\left(g\right)}$

The real intake per day according to the daily intake was calculated based on the serving size indicated on the label: starting formulas: 751 mL/day, follow-up formulas: 675 mL/day, growing-up formulas: 540 mL/day, extra-care formulas: 770 mL/day, cereals: 44 g/day, cornflakes: 30 g/day, biscuits: 16 g/day, puree food: 361 g/day:

$$\mathrm{Real}\;\mathrm{intake}\;\mathrm{per}\;\mathrm{day}=\frac{\%\mathrm{DV}\;\times \;\mathrm{serving}\;\mathrm{size}\;\left(\mathrm{from}\;\mathrm{the}\;\mathrm{label}\right)}{100\;\left(\mathrm{reference}\;\mathrm{intake}*\right)}$$

* Reference intake is defined as 100 mL for infant formulas and 100 g for baby food products.

## 3. Results

A total of 41 infant formulas and 76 baby food products available in the Lebanese market were identified between April and May 2021. Tables S1 and S2 provide a summary of the nutrient content of the tested products.

### 3.1. Total Energy, Macronutrients, Ash, Moisture, and Chloride Composition of Infant Formulas and Baby Food Products

#### 3.1.1. Total Energy

The energy content in infant formulas ranged from 432 to 508 kcal/100 g. The highest content of energy was reported in the starting formulas with a mean ± SD of 489.5 ± 14.4 kcal/100 g. Yet, growing-up formulas had the lowest calorific value of 453.7 ± 13.86 kcal/100 g. As for baby food products, the content of total energy ranged between 31 and 472 kcal/100 g. Among biscuits, cereals, and cornflakes, the highest mean value was detected in biscuits (437.28 ± 26.1 kcal/100 g). As for the pureed foods, milk-based puree had the maximum calorific content of 92.5 ± 4.94 kcal/100 g.

#### 3.1.2. Total Fat

**Figure 1:**
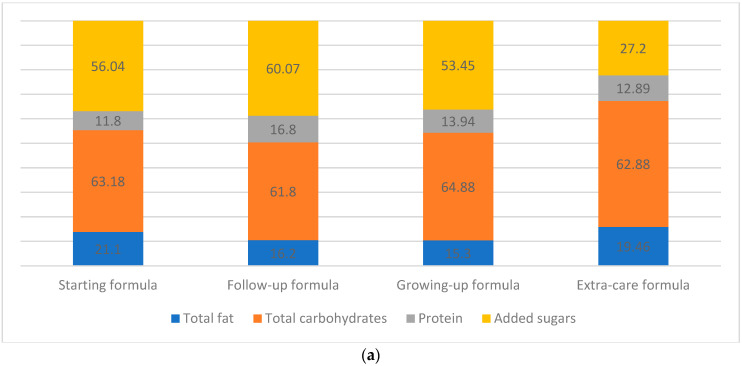

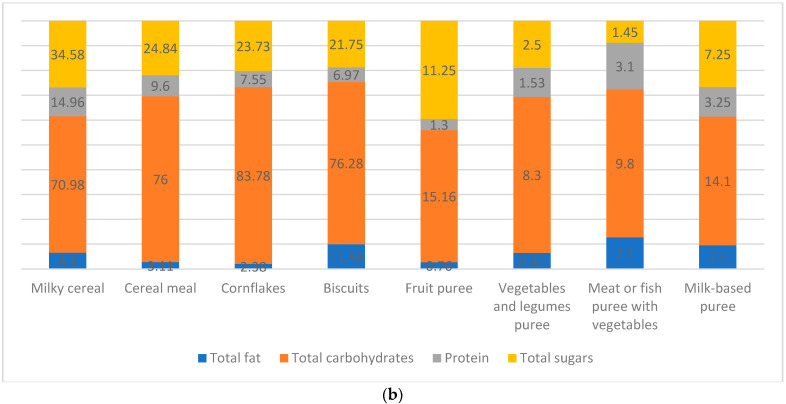
(**a**) Percentage of total energy, macronutrient, and added sugar distribution in 100 g of infant formula: starting formulas (0–6 months); follow-up formulas (6–12 months); growing-up formulas (1–3 years); and extra-care formulas (0–12 years). (**b**) Percentage of total energy, macronutrient, and added sugar distribution in 100 g of baby food products that targeted children aged 6 months and plus.

The total fat content in infant formulas ranged from 6.4 to 25.2 g/100 g. Starting formulas had the highest fat content of 21.1 ± 2.8 g/100 g. The lowest content was seen among growing-up formulas with a mean value of 15.3 ± 2.9 g/100 g (Figure 1a). Moreover, the amount of total fat in baby food products had a minimum level of 0.1 g/100 g and a maximum level of 17.3 g/100 g. Among biscuits, cereals, and cornflakes, biscuits are reported to have the highest mean value of 11.58 ± 4.74 g/100 g. As for the pureed foods, milk-based puree had the highest fat content (2.6 ± 1.13 g/100 g) (Figure 1b).

##### Fatty Acids

**Figure 2:**
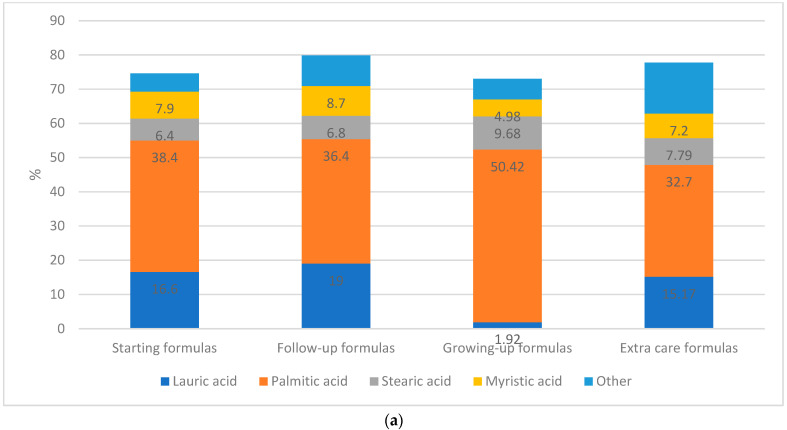

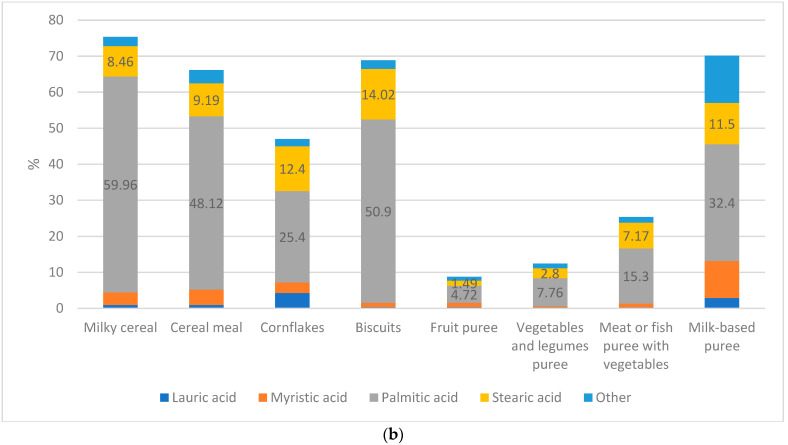
(**a**) Percentage of saturated fatty acids distribution (from total fatty acids) in infant formula: starting formulas (0–6 months); follow-up formulas (6–12 months); growing-up formulas (1–3 years); and extra-care formulas (0–12 years). (**b**) Percentage of saturated fatty acids distribution (from total fatty acids) in baby food products that targeted children aged 6 months and plus. Other: Butyric acid, Caproic acid, Caprylic acid, Capric acid, Undecanoic acid, Tridecanoic acid, Pentadecanoic acid, Heptadecanoic acid, Arachidic acid, Heneicosanoic acid, Behenic acid, Tricosanoic acid, Lignoceric acid.

The highest content of total saturated fatty acids was reported in the follow-up formulas (79.85 g/100 g). Among all fatty acids, palmitic acid (C16:0) accounted for the greatest proportion with a range of 11.4 and 68.2 g/100 g; growing-up formulas contain the highest amount with a mean ± SD of 50.42 ± 9.30 g/100 g (Figure 2a). As for baby food products, the saturated fatty acid content had a minimum and maximum level of 8.78 g/100 g in fruit puree and 75.38 g/100 g in milky cereal, respectively. Palmitic acid (C16:0) contributed as well for the highest amount of total fatty acid in infant foods, except for fruit puree and vegetable and legume puree (Figure 2b).

Monounsaturated fatty acids’ content was between 17.4 g/100 g in the follow-up formulas and 26.28 g/100 g in the starting formulas. Oleic acid (C18:1) had the highest percentage among total fatty acids, where extra-care formulas contained the maximal amount of 16.4 ± 10.27 g/100 g. Baby food products reported a monounsaturated fatty acids’ amount ranging from 5.19 g/100 g in the fruit puree and 52.5 g/100 g in the meat or fish puree with vegetables. Oleic acid (C18:1) accounted as well for the greatest amount of total fatty acid in infant foods with the highest level detected in meat or fish puree with vegetables (49.69 ± 7.18 g/100 g).

The content of polyunsaturated fatty acids in infant formulas was between 2.16 g/100 g in the follow-up formulas and 2.67 g/100 g in the growing-up formulas. Among all fatty acids, linolenic acid (C18:3) accounted for the greatest proportion with a range from 0.1 to 2 g/100 g; growing-up formulas contained the highest amount with a mean ± SD of 1.31 ± 0.56 g/100 g. Polyunsaturated fatty acids in baby food products were found within a range of 1.53–22.5 g/100 g. The percentage of linoleic acid (C18:2) was detected to be high among the total fatty acids; meat or fish puree with vegetables had the highest level (17.05 ± 9.1 g/100 g).

Trans fatty acids’ content in infant formulas had a minimum level of 0.25 g/100 g in the starting formulas and a maximum level of 0.59 g/100 g in the growing-up formulas. Further, among all baby food products, milk-based puree accounted for the highest level of trans fatty acids (1.65 g/100 g).

#### 3.1.3. Total Carbohydrates

The total carbohydrate content in infant formulas was 47.8–75.4 g/100 g. The highest content was detected in growing-up formulas with a mean ± SD of 64.88 ± 8.92 g/100 g, while follow-up formulas accounted for the lowest amount (61.8 ± 2.9 g/100 g) (Figure 1a). As for baby food products, the content of total carbohydrates was between 6.3 and 90.2 g/100 g. Among biscuits, cereals, and cornflakes, the highest mean value was detected in cornflakes (83.78 ± 4.12 g/100 g). As for the pureed foods, fruit puree had the maximum carbohydrate content (15.16 ± 3.59 g/100 g) (Figure 1b).

##### Added Sugars

**Figure 3:**
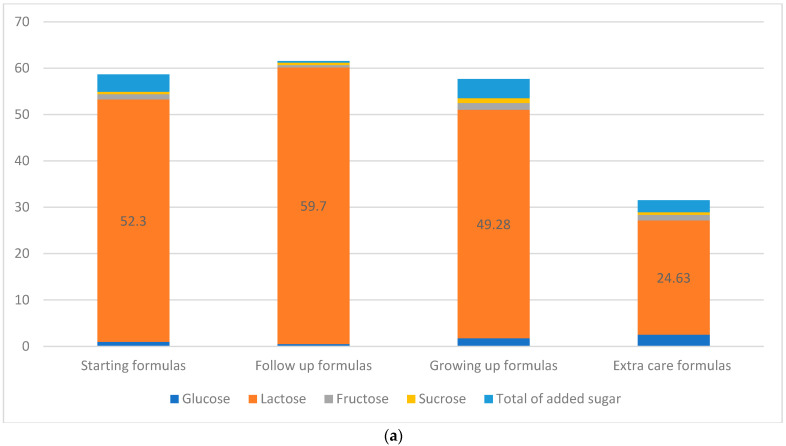

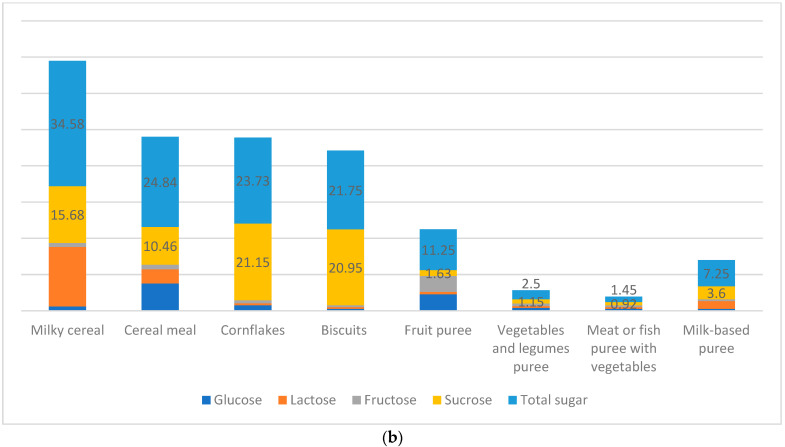
(**a**) Percentage of lactose and added sugar distribution in 100 g of infant formulas: starting formulas (0–6 months); follow-up formulas (6–12 months); growing-up formulas (1–3 years), and extra-care formulas (0–12 years). (**b**) Percentage of added sugar distribution in 100 g of baby food products that targeted children aged 6 months and plus.

The total added sugar content in infant formulas was detected to be between 0.37 g/100 g in follow-up formulas and 4.17 g/100 g in growing-up formulas. Among total added sugars, glucose was the predominant added sugar in infant formulas, where extra-care formulas accounted for the highest concentration (2.53 ± 2.62 g/100 g), followed by fructose that was the highest in the growing-up formulas (1.48 ± 1.27 g/100 g) (Figure 3a). Additionally, the amount of total sugar among biscuits, cereals, and cornflakes, and milky cereals had a maximum level of 34.58 ± 0.48 g/100 g. As for the pureed food, fruit puree had the maximum total sugar content (11.25 ± 4.05 g/100 g). From total sugars, sucrose was most predominant in infant foods, of which cornflakes contributed to the highest proportion of 21.15 ± 8.74 g/100 g (Figure 3b).

#### 3.1.4. Protein

Protein content in infant formulas was 5.3–63.7 g/100 g. The highest content was detected in follow-up formulas with a mean ± SD of 16.8 ± 3.16 g/100 g, while staring formulas accounted for the lowest amount (11.8 ± 1.57 g/100 g) (Figure 3a). As for baby food products, the protein content was reported to be between 0.3 and 17.6 g/100 g. Among biscuits, cereals, and cornflakes, the highest mean value was detected in milky cereals (14.96 ± 1.54 g/100 g). As for the pureed food, milk-based puree accounted for the highest protein content (3.25 ± 0.07 g/100 g) (Figure 3b).

### 3.2. Compliance of the Measured Values of Infant Formulas and Baby Food Products with Codex, EFSA, and Libnor Regulations

Table 1 compares some of the measured products and their conversions to the standards established by the Codex Alimentarius, EFSA, and Libnor.

The follow-up formulas (3.51 g/100 kcal) and growing-up formulas (3.37 g/100 kcal) had total fat levels below the recommended limits of EFSA (4.4–6 g/100 kcal). Additionally, all baby food products’ fat content was lower than the regulations set by Codex Alimentarius (at least 20% of total energy from fat) and Libnor (10–25 g/100 g), with the exception of meat or fish puree with vegetables and milk-based puree that was consistent with the Codex regulations only. The linoleic and alpha linolenic acid content in the infant formulas was below the recommendations except for the alpha-linolenic content in starting formulas (53.16 mg/100 kcal). Further, all baby food products had linoleic acid levels that were below the reference range, except for the meat or fish puree with vegetables (492.26 mg/100 kcal). As for the carbohydrate content, it exceeded EFSA’s regulations (14 g/100 kcal) in the growing-up formulas (14.3 g/100 kcal). Furthermore, the protein content (3.65 g/100 kcal) of the growing-up formulas (3.07 g/100 Kcal) and extra-care formulas (2.69 g/100 kcal) exceeded EFSA’s regulations (1.8–2.5 g/100 Kcal). Moreover, the protein content of the baby food products was below the regulations except for cereal meal.

A more detailed description of the above findings can be found in the Supplementary Material Table S3.

### 3.3. Contributions to Daily Values

The contributions to daily values are shown in Table 2. The total fat content of all infant formulas, cereal meals, cornflakes, and pureed foods was low in which their contribution to the DV was below 10%. Moreover, the saturated fatty acids in the growing-up formulas contributed to 85% of the total daily intake. Furthermore, the trans fatty acid %DV was low in the growing-up formulas (8.2%) and all the baby food products. Likewise, the carbohydrate content was low (<10%) in all the infant formulas and vegetable and legume puree, good (10–19%) in fruit puree, meat or fish puree with vegetables and milk-based puree, and high (>20%) in biscuits, cornflakes, cereal meals, and milky cereals. Hence, the contribution of added sugar to the daily intake was reported to be 155.5%, 162.4%, and 104.7% in growing-up formulas, fruit puree, and milk-based puree, respectively. Additionally, the %DV of protein content was higher than 10% in all the infant formulas and greater than 20% in all the baby food products except for fruit puree (11.8%) and vegetable and legume puree (13.9%). Further, the contribution of protein to the daily intake was seen to be 131%, 139.05%, 147.07%, 101.8%, and 106.5% amongst starting formulas, follow-up formulas, extra-care formulas, meat or fish puree with vegetables, and milk-based puree, respectively.

### 3.4. Nutrient Content and Labeling Discrepancies in Infant Formulas and Baby Food Products

When compared to the content of each product’s nutrition facts label, it was discovered that the majority of the products had inconsistencies in reflecting the real nutrient content and some of them had undeclared values. Most products showed a deviation from 0.5% to 46% between the label and the laboratory values (Table 3).

Referring to the energy content, a non-conformity was seen among the starting formulas, extra-care formulas, cereal meal, fruit puree, and meat or fish puree with vegetables with a deviation of 12.6%, 17.9%, 7.3%, 8.23%, and 5.52%, respectively. This difference was statistically significant. Moreover, the content of total fat was significantly different in all infant formulas, cornflakes, and biscuits with a deviation ranging from 0.69% to 6.04%. The labeled and tested content of saturated, mono-, and polyunsaturated fatty acids showed discrepancies between 13.45% and 39.6% in all infant formulas. As for the content of saturated fatty acids in all baby food products, a non-conformity was detected, except for fruit puree, vegetable and legume puree, and milk-based puree. Furthermore, the measured and labeled values of all infant formulas, biscuits, fruit puree, vegetable and legume puree, meat or fish puree with vegetables were found to differ significantly in terms of total carbohydrates; this difference ranged from 1.5% to 11.98%. Further, a statistically significant non-conformity was detected between the measured and labeled lactose content in all breast milk substitutes except for starting formulas (range: 6.82–8.94%). More notably, the fructose and sucrose content were not labeled in all infant formulas. A deviation in the protein content ranging from 0.25% to 0.95% was statistically significant among the starting formulas, cereal meal, cornflakes, fruit puree, and vegetable and legume puree.

## 4. Discussion

Our paper is the first to provide an overview of the energy and nutrient composition of 117 commercially available infant formulas and baby food products in the Lebanese market. Based on our findings, starting formulas and biscuits were found to have the highest content of energy (489.5 ± 14.4 kcal/100 g, 437.28 ± 26.1 kcal/100 g, respectively) and total fat (21.1 ± 2.8 g/100 g, 11.58 ± 4.74 g/100 g, respectively). Moreover, saturated fatty acid content was detected to be the highest in follow-up formulas (79.85 g/100 g) and milky cereals (75.38 g/100 g). Among all saturated fatty acids, palmitic acid (C16:0) accounted for the greatest proportion. Further, the content of trans fatty acids accounted for a maximum level of 0.59 g/100 g in growing-up formulas and 1.65 g/100 g in milk-based puree. Regarding the carbohydrate content, growing-up formulas and cornflakes reported the highest amount (64.88 ± 8.92 g/100 g, 83.78 ± 4.12 g/100 g, respectively). Additionally, growing-up formulas and milky cereals had the highest total added sugar content (4.17 g/100 g, 34.58 ± 0.48 g/100 g, respectively). Moreover, glucose and sucrose were the predominant added sugars in infant formulas, while sucrose was the main added sugar in baby food products.

Our study also found that the fat composition of the majority of infant formulas and baby food products was below the regulations, whereas the protein content was lower in most baby food products and higher than the regulations in most infant formulas. Additionally, our data showed that the majority of the products had discrepancies in reflecting the real nutrient content when compared to the nutrition facts label.

Our results stated also that the contribution to the daily value for the saturated fatty acids, added sugars, and protein exceeded the daily recommended intake for most infant formulas and baby food products.

### 4.1. Comparison of the Composition of Infant Formulas and Baby Food Products with Regional Data

In our study, the minimum and maximum levels of calories detected in infant formulas ranged from 432 to 508 kcal/100 g, higher than the range reported in Pakistan (428–473 kcal/100 g) [20]. Moreover, our findings showed that starting formulas had the highest content of energy (489.5 ± 14.4 kcal/100 g) and growing-up formulas had the lowest (453.7 ± 13.86 kcal/100 g), similar to that reported in a Saudi study (481.68 and 467.06 kcal/100 g, respectively) [21].

The measured fat content detected in infant formulas in our study ranged from 6.4 to 25.2 g/100 g, lower than that detected in Egypt (18.7–26.7 g/100 g) [22], Pakistan in 1985 (18.2–27 g/100 g) [20], and Pakistan in 2021 (9.82–26.63 g/100 g) [23], and higher than the fat content analyzed in Kuwait (0.23–5.2 g/100 g) [24]. Further, our findings were in line with a Saudi study that revealed a higher fat content in starting formulas (23.27 g/100 g) [21]. As for baby food products, our fat content detected in fruit puree (0.1–2.7 g/100 g) and vegetable and legume puree (0.2–1.6 g/100 g) was the lowest; this was in line with an Egyptian study where the fruit, vegetable, and legume purees were 0.15–2.18 g/100 g [25]. Among all fatty acids in infant formulas, palmitic acid (C16:0) was the dominant saturated fatty acid with a range from 11.4 to 68.2 g/100 g, and oleic acid accounted for the highest proportion among monounsaturated fatty acids (5–37.1 g/100 g); our findings were in line with Sudan [26] and Kuwait [27].

In infant formulas, our results showed a total carbohydrate content with an average of 63.18 g/100 g, higher than that of Egypt in 2014 (53.04 ± 2.31 g/100 g) [28], Saudi Arabia (55 g/100 g) [21], and Pakistan (52 g/100 g) [20], and lower than that of Egypt in 2016 (64.92 g/100 g) [22]. Moreover, our carbohydrate content in milky cereals (70.98 ± 3.06 g/100 g) was lower than that declared in Pakistan (74.6 g/100 g) [20].

Protein content in infant formulas was detected with a mean of 13.85 g/100 g, close to the composition detected in a Kuwaiti Study (2016) (13.48 g/100 g) [24], higher than that analyzed in Egypt (2014) (11.07 g/100 g) [28], Egypt (2016) (8.88 g/100 g) [22], and Pakistan (2021) (12.61 g/100 g) [23], and lower than that assessed in Saudi Arabia (15.17 g/100 g) [21] and Pakistan (1985) (19.65 g/100 g) [20]. As for baby food products, the highest mean value was detected in milky cereals (14.96 ± 1.54 g/100 g); this finding was higher than the protein composition detected in Pakistan (12.5 g/100 g) [20]. Results are presented in Table S4.

### 4.2. Comparison of the Composition of Infant Formulas and Baby Food Products with International Data

The findings of our study revealed a level of calories of 437.28 kcal/100 g in biscuits, lower than that reported in Turkey (468.3 kcal/100 g) [29]. In a study conducted in the United Kingdom, the level of energy was lower than that detected in our study for meat or fish puree with vegetables (71.1 kcal/100 g) [30]. Furthermore, our results were very close to a study conducted in Turkey (386 kcal/100 g) for cereal meal (399.7 kcal/100 g) [29].

The mean fat content detected in infant formulas in our study was 18 g/100 g, lower than that detected in Italy (26.2/100 g) [31]. As for baby food products, the fat content detected in biscuits (11.58 g/100 g) and cereal meal (3.11 g/100 g) was lower than that detected in Turkey (19.7 g/100 g; 4.3 g/100 g, respectively) [29]. Our findings were in line with a British study that revealed a similar fat content in meat-based puree (2.1 g/100 g) [30], whereas a higher fat content (2.5 g/100 g) was reported for vegetable and legume puree compared to our results (0.85 g/100 g) [30]. Further, the saturated fatty acid content in our study in infant formulas (starting formula: 74.59 g/100 g; extra-care formula: 77.72 g/100 g) was much higher than that detected in Brazil (starting formula: 42.3 g/100 g; extra-care formulas: 41.9 g/100 g, respectively) [32], Spain (37.31 g/100 g and 35.9 g/100 g) [33,34], and the USA (41.05 g/100 g) [35]. Additionally, in the current study, palmitic acid content in infant formulas was much higher than that reported in Brazil (starting formula: 19.18 g/100 g; extra-care formula: 17 g/100 g) [32], Cote d’Ivoire (24.74 g/100 g) [36], Spain (23.09 g/100 g) [33], and the USA (16.2 g/100 g) [35]. The measured trans fatty acids in infant formulas (0.38 g/100 g) were higher than that reported in Spain (0.03 g/100 g and 0.33 g/100 g) [33,34], and lower than that found in the USA (1.3 g/100 g) [35].

In infant formulas, our results showed a total carbohydrate content with an average of 63.18 g/100 g, higher than that of Italy (56.3 g/100 g). Moreover, our carbohydrate content in baby food products was higher than that of Turkey (biscuits: 76.28 g/100 g vs. 67.3 g/100 g) [29], the United Kingdom (meat or fish puree with vegetables: 9.8 g/100 g vs. 7.4 g/100 g; vegetable and legume puree: 8.3 g/100 g vs. 7.4 g/100 g) [30]. However, for cereal meal, our results were in line with a Turkish study (76 g/100 g vs. 76.2 g/100 g) [29]. As for added sugar, their content in biscuits (21.75 g/100 g) was higher than that detected in Canada (19 g/100 g) [37] and lower than that detected in Turkey (31.4 g/100 g) [29], whereas their content in cereal meal (24.84 g/100 g) was higher than both studies conducted in Spain [38] and Turkey [29].

Protein content in infant formulas was detected with a mean of 13.85 g/100 g higher than that analyzed in Italy (10.9 g/100 g) [31]. As for baby food products, the protein content was in alignment with a British study for meat or fish puree with vegetables (3.1 g/100 g vs. 3.2 g/100 g) [30], but higher for vegetable and legume puree (1.53 g/100 g vs. 2 g/100 g) [30]. Results are presented in Table S4.

### 4.3. Associated Health Risks of Infant Formulas and Baby Food Products

According to Codex Alimentarius, starting formulas containing hydrolyzed protein or casein from cows’ milk should ideally include lactose and glucose polymers as their primary sources of carbohydrates [39]. The tested samples were compliant with this notion of Codex, where lactose was found to be the principal constituent. Additionally, in the current study, the majority of analyzed infant formula samples contained added sugars (fructose, glucose, sucrose) in different proportions. As per ESPHGAN, fructose should not be added to infant formulas, used during the first six months of a baby’s life [40], because of the risk of life-threatening symptoms in children with undiagnosed hereditary fructose intolerance [39]. Fructose was detected in all the starting formula samples; however, it was present in a higher concentration in four starting formula samples (3.7, 3.7, 5.3, and 4.8 g/100 g). This is of utmost importance because the samples are meant to be consumed by infants between the ages of 0 and 6 months. Furthermore, the addition of sucrose as an ingredient should be avoided in infant formula unless absolutely necessary [39], whereas, starting, follow-up formulas, and growing-up formulas included trace levels of sucrose, demonstrating that this advice was followed in the current study. The cariogenic potential of sucrose-containing solutions in the breast milk substitutes is concerning [41], as sucrose has been recognized as the most cariogenic among added sugars [42]. The contribution of added sugar to the daily intake was reported to be high in infant formulas and baby food products, specifically, in growing-up formulas, fruit puree, milk-based puree, and milky cereals. This is alarming, given the fact that an excessive intake of sugars encourages weight gain, dental caries, and in general, the development of noncommunicable diseases (NCDs), including obesity [15]. Low- and middle-income countries are currently dealing with the so-called double burden of malnutrition. Although undernutrition is taking the vast majority, a significant growth in obesity and overweight cases associated with NCDs is very common in children under 5 years of age [43]. This is clearly related to the children’s nutritional status, which includes exposure to high fat, calorie dense, and micronutrient-deficient meals [43]. Hence, if the baby food industry is truly dedicated to improving the health of children, the formulation of such foods should be adjusted to incorporate less sugar than is currently available in infant products.

There are no regulations in Codex Alimentarius and EFSA that specify the maximum level for saturated fatty acids, specifically palmitic acid. Notably, the highest proportion among all saturated fatty acids accounted for palmitic acid, in infant formulas and baby food products. Different studies have revealed its negative effect on infant health. A systematic review showed that the use of palmitic acid is associated with the decreased intestinal absorption of fat and calcium, hence a lower bone mass density [44,45]. This can lead to a higher risk of osteoporosis and childhood fractures in the future [46]. Further, a meta-analysis of randomized clinical trials indicated that palm-fed infants have harder stools [45,46,47]. Moreover, a high palmitic acid and low linoleic acid fractions are associated with myocardial infarction, stroke, left ventricular hypertrophy, and metabolic syndrome. It is also recognized that a large amount of saturated fat causes insulin resistance, glucose intolerance, metabolic syndrome, and low-grade inflammation [48].

The protein content in the majority of the analyzed samples of infant formulas and baby food products was non-compliant with the regulations. The contribution of protein to the daily intake was seen to be high among starting, follow-up, extra-care formulas, meat or fish puree with vegetables, and milk-based puree. Referring to the literature, an excessive amount of protein at a young age was linked to a lower calcium intake; additionally, non-breastfed infants who are dependent on high protein infant formulas acquire rapid increases in their body weight and fat mass, which increase the risk of overweight and obesity, diabetes, hypertension, and cardiovascular diseases later in life [49].

### 4.4. Nutrient Content and Labeling Discrepancies in Infant Formulas and Baby Food Products

There are scant data on packages about the types of fats and added sugars found in infant formulas and baby food products. When comparing the nutrition facts label with our measured values, it was determined that the majority of the items had inconsistencies in representing the actual nutrient content, and several of them had undeclared values. The current study’s findings coincided with an Emirati study [41], an Egyptian study [22], and a Spanish study [38], in which a range of differences was noted.

The disparity between the labeled and analyzed glucose levels in infant formulas was detected to be slight, ranging between 0.5% and 2%, while some of these measurements had more or less detected glucose than was labeled. Furthermore, there was no indication of the amount of fructose or sucrose contained in the infant formulas on the packaging, despite the fact that both of these sugars were detected. Regarding the baby food products, the total amount of added sugar was not declared. Further, there was no indication of the level of trans fatty acids on the labels, despite the fact that the vast majority of analyzed infant formulas and baby food products contained trans fatty acids (all but three). As for saturated, mono-, and polyunsaturated fatty acids, the discrepancy between the declared and measured value was high, ranging between 2.58% and 45.6%. These findings are rather frightening because, despite the fact that the total carbohydrate and fat levels were disclosed, the sugars, fatty acids, and their respective values were not included on the labels. This can lead to confusion for the parents who purchase these products.

### 4.5. Strengths and Limitations

To the best of our knowledge, this study is the first to investigate the nutrient composition of infant formulas and baby food products in a detailed manner, in Lebanon and the Middle East. However, due to the shortages of some items on the Lebanese market, only available infant formulas and baby food products were selected.

## 5. Conclusions

The current study provides information on the nutrient content of foods that are intended for infants and young children in Lebanon. The findings of this study are very alarming, revealing a high content of proteins and added sugars in the majority of infant formulas and baby food products. A non-compliance to the regulations and discrepancies in reflecting the real nutrient content on the nutrition fact label of these products was seen as well. Of utmost concern is that some nutrients exceeded the daily recommended intake. The analysis of the nutritional composition of infant formulas and baby foods that are available on the Lebanese market has brought to light some aspects that require careful evaluation in order to improve the feeding practices of infants and young children. This is a strong call to action for policymakers and high authorities in Lebanon to ensure that children can have the best, healthy, and nutritious start in life.

## Figures and Tables

**Table 1:** Comparison between the measured values of nutrient content in infant formulas with the regulations.

	Carbohydrates	Codex ^/ EFSA +/Libnor Regulations	Protein	Codex/ EFSA/Libnor Regulations	Total Calories from Protein	Codex/ EFSA/Libnor Regulations	Total Fat	Codex/ EFSA/Libnor Regulations	Total Calories from Fat	Codex/ EFSA/Libnor Regulations
Follow-up formulas	-	-	3.65 g/100kcal *	Exceeding EFSA	-	-	-	-	-	-
Growing-up formulas	14.3 g/100kcal *	Exceeding EFSA	3.07 g/100kcal *	Exceeding EFSA	-	-	-	-	-	-
Extra-care formulas	-	-	2.69 g/100kcal *	Exceeding EFSA	-	-	4.06 g/100kcal *	Below Codex	-	-
Milky cereal	-	-	14.96	Below Libnor	-	-	8.4 g/100g	Below Libnor	75.6 Kcal **	Below Codex
Cereal meal	-	-	-	-	-	-	3.11 g/100g	Below Libnor	28 Kcal **	Below Codex
Cornflakes	-	-	7.55 g/100kcal *	Below Libnor	-	-	2.38 g/100g	Below Libnor	21.42 Kcal **	Below Codex
Biscuits	-	-	6.97 g/100kcal *	Below Libnor	-	-	-	-	-	-
Fruit puree	-	-	1.3 g/100kcal *	Below Libnor	-	-	0.76 g/100g	Below Libnor	6.84 Kcal **	Below Codex
Vegetable and legume puree	-	-	1.53 g/100kcal	Below Libnor	-	-	0.85 g/100g	Below Libnor	7.65 Kcal **	Below Codex
Meat or fish puree with vegetables	-	-	3.1 g/100kcal *	Below Libnor	12.4 Kcal ***	Exceeding Codex	2.1 g/100g	Below Libnor	-	-
Milk-based puree	-	-	3.25 g/100kcal *	Below Libnor	-	-	2.6 g/100g	Below Libnor	-	-

Values are mean ± SD; Min: minimum; Max: maximum; C: concentration; ND: not determined. * Calculated in mg or g/100 kcal to compare with the regulations: (measured value (g/100 g)/total calories (100 g)) × 100. ** Calculated to compare with Codex (based on fat recommendation of at least 20% of total energy). *** Calculated to compare with Codex (based on protein recommendation of 6–15% of total energy). + https://www.legislation.gov.uk/eur/2016/127/contents?view=plain. ^ https://www.fao.org/fao-who-codexalimentarius/sh-proxy/en/?lnk=1&url=https%253A%252F%252Fworkspace.fao.org%252Fsites%252Fcodex%252FStandards%252FCXS%2B72-1981%252FCXS_072e.pdf. ^ https://www.fao.org/fao-who-codexalimentarius/sh-proxy/en/?lnk=1&url=https%253A%252F%252Fworkspace.fao.org%252Fsites%252Fcodex%252FStandards%252FCXS%2B156-1987%252FCXS_156e.pdf (accessed on 3 August 2022).

**Table 2:** The percentage of daily value of infant formulas and baby food products for the amount of total fat, saturated fatty acids, trans fatty acids, total carbs, added sugar, and protein based on daily recommended amounts.

	Percentage of Contribution to the Daily Value						Percentage of Real Intake per Day According to the Daily Intake (Label)					
	Total Fat	SFA	TFA	Total Carbs	Added Sugar	Protein	Total Fat	SFA	TFA	Total Carbs	Added Sugar	Protein
Infant formulas (100 mL) +^												
Starting formulas (0–6 months)	9.5	NA	NA	8.9	NA	17.5	71.3	NA	NA	66.8	NA	131
Follow-up formulas (6–12 months)	7.3	NA	NA	8.7	NA	20.6	49.3	NA	NA	58.7	NA	139.05
Growing-up formulas (1–3 years)	5.2	15.1	8.2	5.8	28.8	14.5	28.1	81.5	44.3	31.3	155.5	78.3
Extra-care formulas (1–3 years)	8.7	NA	NA	8.9	NA	19.1	66.9	NA	NA	68.5	NA	147.07
Baby food products (100 g) +^												
Milky cereals	21.5	63.3	1.68	74.7	138.3	136	9.4	27.8	0.7	32.8	60.8	59.8
Cereal meal	7.9	20.5	1.92	80	99.3	87.2	3.5	9.02	0.8	35.2	43.7	38.3
Cornflakes	6.1	11.2	0.9	88.2	94.9	68.6	1.8	3.3	0.27	26.4	28.5	20.6
Biscuits	29.7	79.7	4.6	80.3	87	63.3	4.75	12.7	0.7	12.8	13.9	10.1
Fruit puree	1.9	0.66	0.06	15.9	45	11.8	6.8	2.4	0.2	57.4	162.4	42.5
Vegetable and legume puree	2.2	1.05	0.51	8.7	10	13.9	7.9	3.8	1.8	31.4	36.1	50.1
Meat or fish puree with vegetables	5.4	5.32	0.93	10.3	5.8	28.2	19.5	19.2	3.3	37.2	20.9	101.8
Milk-based puree	6.6	18.2	3.9	14.8	29	29.5	23.8	65.7	14.07	53.4	104.7	106.5

+ For infant (0–12 months): the daily recommended amount of total fat is 30 g, SFA (saturated fatty acids): not applicable (NA), total carbs: 95 g, added sugar: not applicable (NA), protein: 9.1 g (0–6 months), 11 g (7–12 months) [17]. We did not include the 6–12 months age category while calculating the %DV and the % of real intake in baby food products, because of the lack of daily recommended intakes for SFA, TFA, added sugar, 3 main components that need to be highlighted in the diet of children. ^ For children (1–3 years): the daily recommended amount of total fat is 39 g, total carbs: 150 g, protein: 13 g [17], added sugar: 25 g [18], SFA: 10 g, TFA: 1.1 g [19]. Macronutrient values are based on the reference caloric intake of 1000 kcal for children aged 1–3 years.

**Table 3:** Table summarizing the difference between measured and labeled values.

	Energy	Total Fat	Saturated Fatty Acids	Monounsaturated Fatty Acids	Polyunsaturated Fatty Acids	Trans Fatty Acids	carbohydrates	Total Sugar	Glucose	Lactose	Fructose	Sucrose	Protein
Starting formulas	12.6% *	5% *	33.1% *	21.55% *	15.4% *	NL	7.43% *	NL	0.5%	1.31%	NL	NL	0.95% *
Follow-up formulas	11.86%	4.8% *	39.6% *	NL	NL	NL	5.6% *	NL	NL	8.94% *	NL	NL	2.14%
Growing-up formulas	1.7%	5.04% *	37.4% *	33.77% *	13.45% *	0.65%	11.98% *	8.35%	0.66%	8.65% *	NL	NL	0.47%
Extra-care formulas	17.9% *	6.04% *	31.84% *	19.12% *	14.36% *	0.25%	8.74% *	2.14%	2% *	6.82% *	NL	NL	0.62%
Milky cereal	3.8%	0.52%	36.34% *	NL	NL	NL	2.26%	2.16% *	NL	NL	NL	NL	0%
Cereal Meal	7.3% *	0.33%	45.26% *	NL	NL	NL	3.93%	2.32%	NL	NL	NL	NL	0.58% *
Cornflakes	1.9%	0.69% *	19.49 *	NL	24.63%	0.15	1.62% *	1.66%	NL	NL	NL	NL	0.63% *
Biscuits	3.12%	1.3% *	35.13 *	NL	NL	NL	2.88% *	0.49%	NL	NL	NL	NL	0.51%
Fruit puree	8.23 *	0.2%	18.39%	NL	NL	NL	1.87% *	1.19%	NL	NL	NL	NL	0.46% *
Vegetable and legume puree	4.4%	0.17%	2.58%	NL	NL	NL	1.5% *	0.03%	NL	NL	NL	NL	0.25% *
Meat or fish puree with vegetables	5.52% *	0	8.76% *	NL	NL	NL	1.77% *	0.35%	NL	NL	NL	NL	0.3%
Milk-based puree	8.5%	0.95%	15.73%	NL	NL	NL	0.25%	0.6%	NL	NL	NL	NL	0.6%

Values are mean ± SD; NL: not labeled; * significant at *p*-value < 0.05 for paired sample *t* test.

## Supplementary Materials

The following supporting information can be downloaded at: https://www.mdpi.com/article/10.3390/nu15051200/s1, Table S1: Composition of infant formulas and baby food products; Table S2: Composition of infant formulas and baby food products; Table S3: Comparison between the measured values of nutrient content in infant formulas with the regulations; Table S4: Composition of infant formulas and baby food products from different countries in the world.
