# Supplementary material for: Assessment of the Composition of Breastmilk Substitutes, Commercial Complementary Foods, and Commercial Snack Products Commonly Fed to Infant and Young Children in Lebanon: A Call to Action
Source: Nutrients. 2023 Feb 27;15(5):1200. doi: 10.3390/nu15051200 (PMC10005724; doi:10.3390/nu15051200)
Supplement: Supplementary file 1 [file nutrients-15-01200-s001.zip › nutrients-2143833-supplementary.pdf]

Table S1. Composition of infant formulas and baby food products

	Infant Formulas							
	Starting formulas[1]		Follow-up formulas[2]		Growing-up formulas[3]		Extra care formulas[4]	
	Mean±SD	Range	Mean±SD	Range	Mean±SD	Range	Mean±SD	Range
**Calorific value (kcal/100g)**								
**Total energy**	489.5±14.4	461-508	460.3±6.6	456-468	453.7±13.86	432-468	478.2±24.8	408-508
**Macronutrients (%)**								
**Total fat**	21.1±2.8	14.9-52	16.2±1.4	15-40.5	15.3±2.9	10.8-20	19.46±4.66	6.4-25.2
**SFA*+**	74.59	-	79.85	-	73.01	-	77.72	-
C4:0	0.62±1.23	0.1-4.1	3.6±5.7	0.2-10.3	2.52±3.01	<0.1-7.9	1.25±1.5	<0.1-4.5
C6:0	0.27±0.14	0.1-0.6	0.26±0.05	0.2-0.3	0.47±0.41	<0.1-1.3	0.3±0.11	0.2-0.6
C8:0	1.4±1.08	0.2-4.1	1.9±0.05	1.9-2	0.4±0.24	<0.1-0.7	6.3±10.4	0.6-36.8
C10:0	2.03±0.74	1.1-3.4	2.3±0.4	2-2.8	1.08±1.44	<0.1-4.1	5.8±7.6	1.1-25.5
C11:0	<0.1	-	<0.1	-	≤0.1	-	<0.1	-
C12:0	16.6±5.6	7.4-26.6	19±2.5	16.3-21.4	1.92±2.04	0.4-6.5	15.17±8.1	0.3-26.6
C13:0	0.11±0.04	0.1-0.2	≤0.1	-	0.11±0.035	0.1-0.2	0.17±0.09	<0.1-0.3
C14:0	7.9±1.8	4.9-11.2	8.7±0.8	8-9.6	4.98±5.25	1.4-16.4	7.2±3.2	0.4-11.8
C15:0	0.14±0.11	0.1-0.5	0.13±0.05	0.1-0.2	0.51±0.52	0.2-1.6	0.19±0.27	<0.1-1.2
C16:0	38.4±8.3	17.8-52	36.4±3.7	33.1-40.5	50.42±9.30	39.5-68.2	32.7±11.08	11.4-50.8
C17:0	0.14±0.06	0.1-0.3	0.16±0.06	0.1-0.2	0.31±0.18	0.2-0.7	0.15±0.12	0.1-0.6
C18:0	6.4±1.9	0.7-8.9	6.8±1.1	5.8-8	9.68±2.09	7.6-14	7.79±2.32	4.8-12.9
C20:0	0.2±0.07	0.1-0.3	0.2±0.06	0.2-0.3	0.34±0.19	0.1-0.6	0.21±0.07	0.1-0.3
C22:0	<0.1	-	-	-	-	-	<0.1	-
C23:0	0.18±0.07	<0.1-0.3	0.2±3E-17	-	0.17±0.09	<0.1-0.3	0.19±0.07	<0.1-0.3
C24:0	-	-	-	-	-	-	<0.1	-
**MUFA*+**	26.28	-	17.4	-	24.82	-	21.41	-
C14:1	0.11±0.03	0.1-0.2	0.1±2E-17	-	0.27±0.29	<0.1-0.9	0.2±0.36	<0.1-1.3
C15:1	≤0.1	-	<0.1		0.14±0.07	<0.1-0.3	0.11±0.04	<0.1-0.3
C16:1	4.1±1.42	1.7-6	4.5±0.2	4.3-4.7	5.62±3.74	0.6-10.7	3.76±2.25	<0.1-7.8
C17:1	≤0.1	-	<0.1	-	0.17±0.17	<0.1-0.6	0.11±0.04	<0.1-0.3
C18:1	17.4±9.6	5-36.6	11.9±1.3	10.7-13.3	17.5±6.39	9.3-27.7	16.4±10.27	1.6-37.5
C20:1	0.7±0.16	0.5-1.1	0.7±0.15	0.6-0.9	1.02±0.45	0.1-1.6	0.7±0.15	0.5-1

C22:1	-	-	-	-	<0.1	-	0.13±0.05	<0.1-0.2
**PUFA*+**	2.46	-	2.16	-	2.67	-	2.53	-
C18:2	0.4±0.3	0.1-1.2	0.3±0.06	0.3-0.4	0.2±0.12	0.1-0.4	0.6±0.84	0.1-3.2
C18:3	1.25±0.26	0.9-1.8	1.1±0.26	0.9-1.4	1.31±0.56	0.6-2	0.84±0.5	0.1-1.9
C20:3	0.5±0.22	0.3-1.2	0.6±0.21	0.5-0.9	1.01±0.85	0.1-2.5	0.58±0.34	0.2-1.4
C20:4	0.16±0.07	0.1-0.3	0.16±0.06	0.1-0.2	0.15±0.11	<0.1-0.4	0.16±0.06	<0.1-0.3
C20:5	0.15±0.07	<0.1-0.2	-	-	-	-	0.35±0.2	<0.1-0.5
**TFA*+**	0.25	-	0.33	-	0.59	-	0.36	-
C18:1t	0.13±0.04	0.1-0.5	0.2±0.17	0.1-0.4	0.32±0.26	0.1-0.9	0.2±0.17	<0.1-0.7
C18:2t	0.13±0.04	<0.1-0.2	0.13±0.06	0.1-0.2	0.27±0.11	0.1-0.4	0.16±0.09	<0.1-0.4
**Total Carbohydrates^**	63.18±4.1	56.9-71.2	61.8±2.9	58.5-64.1	64.88±8.92	47.8-75.4	62.88±4.96	55.5-73.6
**Total sugars**	56.04±11.48	27.5-69.6	60.07±3.3	57.9-63.9	53.45±4.04	47.7-58.8	27.2±22.6	2-67.9
**Added sugars**								
Glucose	0.98±1.27	<0.5-4.8	<0.5	-	1.75±0.88	<0.5-2.8	2.53±2.62	<0.5-9.3
Fructose	1.13±1.47	<0.5-4.8	<0.5	-	1.48±1.27	<0.5-3.5	1.22±1.71	<0.5-5.3
Sucrose	<0.5	-	<0.5	-	1.02±1.39	<0.5-4.2	0.56±0.27	<0.5-1.7
Lactose	52.3±12.4	25.6-69.5	59.7±3.46	57.2-63.7	49.28±4.07	45.7-55.6	24.63±25.34	0.5-64.7
**Protein content**	11.8±1.57	9.6-14.3	16.8±3.16	57.2-63.7	13.94±5.69	5.3-23.7	12.89±2.12	10.3-17.7
**Miscellaneous (%)**								
**Ash**	2.6±0.22	2.3-3.1	3.5±0.15	3.4-3.7	4.05±1.53	2.7-7	3.09±0.68	2-4.5
**Moisture**	1.49±0.43	0.7-2	1.6±0.2	1.4-1.8	1.74±0.58	1-2.7	1.66±0.78	0.5-3.4
**Chlorides, as NaCl**	0.45±0.09	0.2-0.6	0.5±0.1	0.4-0.6	0.58±0.32	0.3-1.1	0.53±0.18	0.2-1

Table S2: Composition of infant formulas and baby food products

	**Baby Food Products**															
	Cereals[5]				Cornflakes[6]		Biscuits[7]		Pureed foods[8]							
	Milky cereal		Cereal meal						Fruit puree		Vegetables and legumes puree		Meat or fish puree with vegetables		Milk-based puree	
	Mean±SD	Range	Mean±SD	Range	Mean±SD	Range	Mean±SD	Range	Mean±SD	Range	Mean±SD	Range	Mean±SD	Range	Mean±SD	Range

**Calorific value (kcal/100g)**																
**Total energy**	419.2±16.5	390-428	399.7±12.07	389-424	385.8±11.14	372-426	437.28±26.1	408-472	72.83±14.2	53-94	47±13.7	31-66	71.1±8.8	62-94	92.5±4.94	89-96
**Macronutrients (%)**																
**Total fat**	8.4±1.07	7.2-9.4	3.11±3.43	0.6-9	2.38±2.34	0.1-10.5	11.58±4.74	6.5-17.3	0.76±0.88	0.1-2.7	0.85±0.59	0.2-1.6	2.1±0.57	1.2-3.3	2.6±1.13	1.8-3.4
**SFA*+**	75.38	-	66.15	-	47.02	-	68.85	-	8.78	-	12.45	-	25.35	-	70.15	-
C4:0	0.36±0.43	0.1-1.1	0.51±0.78	0-2.3	0.25±0.5	0-2.5	0.14±0.07	0.1-0.3	0.21±0.61	0-2.1	0.03±0.05	0-0.1	<0.1	-	1.05±1.34	<0.1-2
C6:0	0.22±0.21	0.1-0.6	0.4±0.5	0-1.4	0.14±0.23	0-1.1	0.15±0.07	0.1-0.3	0.16±0.46	0-1.6	0.13±0.2	0-0.4	<0.1	-	0.95±0.6	0.5-1.4
C8:0	0.3±0.44	0.1-1.1	0.56±0.71	0-1.9	0.22±0.4	0-1.8	0.5±0.37	0.1-1	0.15±0.39	0-1.3	0.46±0.74	0-1.7	0.19±0.17	0.1-0.7	2.5±3.25	0.2-4.8
C10:0	0.68±0.69	0.1-1.8	0.72±1.06	0-3.6	0.42±0.71	0-2.3	0.24±0.22	0.1-0.7	0.28±0.7	0-2.3	0.15±0.25	0-0.6	0.2±0.15	0.1-0.6	6.6±5.2	2.9-10.3
C11:0	<0.1	-	<0.1	-	<0.1	-	<0.1	-	0.01±0.03	0-0.1	0.03±0.05	0-0.1	0.11±0.05	0.1-0.3	<0.1	-
C12:0	0.92±0.83	0.1-2.3	0.94±1.42	0-4.6	4.28±8.9	0-39	0.3±0.18	0.1-0.6	0.33±0.83	0-2.7	0.25±0.44	0-1.1	0.28±0.32	0.1-1	2.85±1.2	2-3.7
C13:0	<0.1	-	<0.1	-	<0.1	-	0.28±0.11	0.2-0.5	0.01±0.03	0-0.1	0.03±0.05	0-0.1	<0.1	-	<0.1	
C14:0	3.5±2.23	1.8-7.4	4.26±5.07	0-14.1	2.9±4.01	0-16.8	1.25±0.87	0.3-2.3	1.19±3.03	0-10	0.36±0.58	0-1.3	1.06±0.91	0.1-2.8	10.3±4.45	7.2-13.5
C15:0	0.32±0.28	0.1-0.8	0.38±0.39	0-1.4	0.1±0.1	0-0.5	0.14±0.05	0.1-0.2	0.12±0.32	0-1.1	0.06±0.1	0-0.2	0.2±0.17	0.1-0.6	1.05±0.6	0.6-1.5
C16:0	59.96±16.6	42-82.9	48.12±18.06	0-67.7	25.4±16.6	0-53.7	50.9±25.5	19.7-78.2	4.72±12.2	0-40.6	7.76±12.2	0-26.4	15.3±7.49	7.8-31.9	32.4±7.6	27-37.8
C17:0	0.26±0.08	0.2-0.4	0.29±0.2	0-0.7	0.14±0.13	0-0.5	0.18±0.06	0.1-0.3	0.075±0.2	0-0.7	0.08±0.13	0-0.3	0.33±0.37	0.1-1.2	0.55±0.21	0.4-0.7
C18:0	8.46±1.8	6.2-10.4	9.19±5.09	0-16.3	12.4±9.38	0-31.5	14.02±9.06	5.6-31.1	1.49±3.9	0-13.3	2.8±4.45	0-9.9	7.17±5.5	2.2-17.4	11.5±3.8	8.8-14.2
C20:0	<0.1	-	0.34±0.34	0-1.1	0.16±0.18	0-0.7	0.51±0.34	0.1-1	0.03±0.08	0-0.3	0.3±0.46	0-1	0.15±0.09	0.1-0.4	≤0.1	-
C21:0	ND	ND	ND	ND	0.03±0.13	0-0.6	0.02±0.04	0-0.1	ND	ND	ND	ND	ND	ND	ND	ND
C22:0	ND	ND	0.09±0.3	0-1	<0.1	-	ND	ND	ND	ND	ND	ND	0.02±0.04	0-0.1	<0.1	-
C23:0	ND	ND	ND	ND	0.2±0.28	0-0.4	0.12±0.14	0-0.4	ND	ND	ND	ND	0.03±0.06	0-0.2	ND	ND
C24:0	<0.1	-	0.15±0.38	0-1.3	0.08±0.06	0-0.3	ND	ND	0.008±0.02	0-0.1	0.01±0.04	0-0.1	0.01±0.04	0-0.1	ND	ND
**MUFA*+**	21.4	-	21.21	-	24.34	-	27.05	-	5.19	-	19.01	-	52.5	-	23.2	-
C14:1	0.16±0.08	0.1-0.3	0.13±0.1	0-0.4	0.1±0.1	0-0.5	0.14±0.05	0.1-0.2	0.09±0.21	0-0.6	0.03±0.05	0-0.1	0.22±0.29	0.1-1.1	0.7±0.4	0.4-1
C15:1	<0.1	-	0.13±0.15	0-0.6	0.1±0.12	0-0.6	<0.1	-	0.03±0.08	0-0.3	0.05±0.08	0-0.2	0.11±0.05	<0.1-0.3	0.2±0.14	0.1-0.3
C16:1	0.26±0.15	0.1-0.4	3.04±3.34	0-7.7	2.39±3.31	0-9.8	7.6±5.44	0.6-15.8	0.1±0.23	0-0.7	0.08±0.13	0-0.3	1.4±0.79	0.6-3	1.05±0.2	0.9-1.2
C17:1	<0.1	-	0.11±0.09	0-0.4	<0.1	-	<0.1	-	0.025±0.06	0-0.2	0.05±0.08	0-0.2	0.23±0.26	<0.1-1	0.15±0.07	0.1-0.2

C18:1	20.18±17.07	1.4-33.9	16.8±11.09	0-32.4	20.9±16.7	0-60.3	17.9±15.8	2-41.8	4.82±11.98	0-38.5	17.7±27.58	0-57.9	49.69±7.18	39.3-60.2	20.7±6.7	16-25.5
C20:1	0.6±0.12	0.5-0.8	1±0.7	0-2.6	0.75±0.5	0-1.8	1.21±0.61	0.6-1.9	0.13±0.33	0-1.1	1.1±1.76	0-4	0.85±0.94	0.2-3.7	0.4±0.14	0.3-0.5
C22:1	ND	ND	ND	ND	ND	ND	ND	ND	ND	ND	ND	ND	ND	ND	ND	ND
**PUFA*+**	4.06	-	4.36	-	9.86	-	3.94	-	2.58	-	1.53	-	22.25	-	6.05	-
C18:2	3.52±3.78	0.3-9.6	2.8±2.7	0-8.7	8.1±9.5	0-38.6	0.74±1.5	0.1-4.3	2.14±7.17	0-24.9	0.55±0.88	0-2	17.05±9.1	3.6-29.7	4.9±5.15	1-8.8
C18:3	0.34±0.23	0.1-0.7	0.46±0.31	0-1	1.1±1.79	0-8.1	1.25±0.86	0.5-3	0.38±1.29	0-4.5	0.3±0.48	0-1.1	4.45±2.8	0.7-9.4	0.65±0.6	0.2-1.1
C20:2	ND	ND	ND	ND	0.01±0.05	0-0.2	1.04±1.78	0-3.7	0.008±0.02	0-0.1	0.02±0.04	0-0.1	0.05±0.05	0-0.1	<0.1	-
C20:3	0.2±0.14	0.1-0.4	1±1.03	0-3.1	0.54±0.56	0-2.4	0.48±0.88	0.1-2.5	0.06±0.15	0-0.4	0.66±1.03	0-2.1	0.58±0.73	0.1-2.7	≤0.1	-
C20:4	ND	ND	ND	ND	0.01±0.05	0-0.2	0.15±0.13	0-0.4	ND	ND	ND	ND	0.03±0.05	0-0.1	<0.1	-
C20:5	ND	ND	ND	ND	ND	ND	0.28±0.48	0-1	ND	ND	ND	ND	0.05±0.05	0-0.1	<0.1	-
C22:2	ND	ND	0.1±0.33	0-1.1	<0.1	-	ND	ND	ND	ND	ND	ND	0.04±0.09	0-0.3	<0.1	-
**TFA*+**	0.22	-	0.68	-	0.42	-	0.44	-	0.09	-	0.67	-	0.49	-	1.65	-
C18:1t	0.12±0.04	0.1-0.2	0.43±0.36	0-1	0.3±0.38	0-1.3	0.24±0.15	0.1-0.5	0.04±0.1	0-0.3	0.4±0.46	0-0.8	0.34±0.77	<0.1-2.8	1.5±0.5	1.1-1.9
C18:2t	<0.1	-	0.25±0.27	0-1	0.12±0.1	0-0.4	0.2±0.05	0.1-0.5	0.05±0.11	0-0.3	0.27±0.3	0-0.6	0.15±0.11	<0.1-0.5	0.15±0.07	0.1-0.2
**Total Carbohydrates^**	70.98±3.06	67.3-74.6	76±23.68	8.8-90	83.78±4.12	75.6-90.2	76.28±3.1	71.8-79.7	15.16±3.59	11.1-22.5	8.3±2.14	6.3-12.3	9.8±1.7	8.2-15	14.1±1.27	13.2-15
**Total sugars**	34.58±0.48	34.1-35.2	24.84±10.68	1.3-36.1	23.73±10.26	6.2-42.6	21.75±2.4	17.8-24.4	11.25±4.05	3.2-17.5	2.5±2.06	0.8-5.7	1.45±0.64	0.5-2.7	7.25±1.6	6.1-8.4
Glucose	1.16±0.78	0.5-2.4	7.51±13.2	0.5-34.8	1.51±1.49	0-4.6	<0.5	-	4.55±2.47	0.5-8.8	0.8±0.6	0.5-2	<0.5	-	<0.5	-
Fructose	1.12±1.12	0.5-3.1	1.25±1.39	0.5-4.9	0.86±0.65	0.5-2.9	<0.5	-	4.4±2.5	0.5-8.7	0.7±0.3	0.5-1.3	<0.5	-	<0.5	-
Sucrose	15.68±1.67	13-17.4	10.46±8.3	0.5-19.5	21.15±8.74	5.8-35.4	20.95±2.4	16.8-23.8	1.63±1.48	0.5-4.7	1.15±0.9	0.5-2.5	0.92±0.3	0.5-1.4	3.6±0.4	3.3-3.9
Lactose	16.44±1.95	14.4-19.3	3.94±6.38	0.5-16.8	0.54±0.21	0.5-1.5	<0.5	-	0.65±0.35	0.5-1.5	<0.5		0.5±0.14	0.5-1	2.15±1.48	1.1-3.2
**Protein content**	14.96±1.54	13.7-17.6	9.6±3.39	6.1-16.3	7.55±2.3	4.7-12.3	6.97±1.3	5.5-8.9	1.3±0.7	0.3-3.1	1.53±0.7	0.8-2.6	3.1±0.9	2-5	3.25±0.07	3.2-3.3
**Miscellaneous (%)**																
**Ash**	2.74±0.32	2.3-3.1	1.49±0.83	0.4-2.8	1.89±0.62	0.9-3.3	1.37±0.17	1.1-1.6	0.38±0.13	0.2-0.7	0.45±0.13	0.2-0.6	0.46±0.18	0.2-0.9	0.5	-

**Moisture**	2.92±3.02	1-8.3	2.5±1.3	0.3-5.7	4.5±1.4	1.9-6.8	3.78±0.92	2.4-5.1	82.37±3.35	76.6-87	88.8±2.66	85-92	84.4±1.95	79.3-86.3	79.5±0.21	79.4-79.7
**Chlorides, as NaCl**	0.42±0.08	0.3-0.5	0.15±0.12	0.1-0.5	0.88±0.5	0.2-1.7	0.38±0.33	0.1-0.8	0.3±0.3	0.1-1.1	0.48±0.43	0.1-1	0.59±0.55	0.1-2.2	0.8±0.14	0.7-0.9

^including sugars and fibers; *SFA: saturated fatty acid; MUFA: monosaturated fatty acid; PUFA: polyunsaturated fatty acid; TFA: trans fatty acid; [+] % of total fatty acids

Butyric acid (C4:0); Caproic acid (C6:0); Caprylic acid (C8:0); Capric acid (C10:0); Undecanoic acid (C11:0); Lauric acid (C12:0); Tridecanoic acid (C13:0); Myristic acid (C14:0); Pentadecanoic acid (C15:0); Palmitic acid (C16:0); Heptadecanoic acid (C17:0); Stearic acid (C18:0); Arachidic acid (C20:0); Heneicosanoic acid (C21:0); Behenic acid (C22:0); Tricosanoic acid (C23:0); Lignoceric acid (24:0); Myristoleic acid (C14:1); Pentadecenoic acid (C15:1); Palmitoleic acid (C16:1); Heptadecenoic acid (C17:1); Oleic acid (C18:1); Gadoleic acid (C20:1); Erucic acid (C22:1); Linoleic acid (C18:2); Linolenic acid (C18:3); Eicosadienoic acid (C20:2); Eicosatrienoic acid (C20:3); Arachidonic acid (C20:4); Eicosapentaenoic acid (C20:5); Docosadienoic acid (C22:2); Elaidic acid (C18:1t); Linolaidic acid (C18:2t)

A substitute for breastmilk intended for infants aged between: [1]0-6 months; [2]6-12 months ; [3]1-3 years ; [4]for infants with special conditions; [5]Common first solid food to be introduced and can be found in pulverized or ready to eat form ; [6]Can be used as a breakfast cereal for babies; [7]Biscuits that are intended for infants and young children ; [8]Products include fruit purées, vegetable purées, mixed fruit/vegetable purées, meals with meat or fish or chicken, milky desserts, intended for babies and toddlers

Table S3. Comparison between the measured values of nutrient content in infant formulas with the regulations.

	Measured value<br>(Codex^/EFSA+/Libnor regulations)	Items below/exceeding regulations
Starting formula		
Total fat (g/100kcal)*	4.31<br>(4.4-6/3-6/4.4-6)	No
Total SFA (g/100g)	74.59<br>(No regulations)	-
Palmitic acid(g/100g)	38.4<br>(No regulations)	-
Total MUFA(g/100g)	26.28<br>(No regulations)	-
Erucic Acid (g/100kcal)*	0<br>(Max 1/Max 0.4/Max 1)	No
Total PUFA(g/100g)	2.46<br>(No regulations)	-
Linoleic acid (mg/100 kcal)*	16.35<br>(300 -1400/500-1200/300 -1400)	Yes<br>(below all regulations)
Linolenic acid (mg/100kcal)*	53.16<br>(Min 50/50-100/ Min 50)	No
Trans fatty acids (g/100kcal)*	0.12<br>(Max 3/Max 3/Max 3)	No
Carbohydrates (g/100 kcal)*	12.92<br>(9-14/9-14/9-14)	No
Protein (g/100 kcal)*	2.41<br>(1.8-3/1.8-2.5/1.8-3)	No
Chlorides (mg/100kcal)*	91.93<br>(50-160/60-160/50-160)	No
Follow-up formulas		
Total fat (g/100kcal)*	3.51<br>(3-6/4.4-6/3-6)	No
Total SFA(g/100g)	79.85	-

	(No regulations)	
Palmitic acid(g/100g)	36.4<br>(No regulations)	-
Total MUFA(g/100g)	17.4<br>(No regulations)	-
Erucic Acid (g/100kcal)*	0<br>(ND/ Max 0.4/ ND)	No
Total PUFA(g/100g)	2.16<br>(No regulations)	-
Linoleic acid (mg/100 kcal)*	8.68<br>(Min 300/500-1200/Min 300)	Yes<br>(below all regulations)
Linolenic acid (mg/100kcal)*	36.93<br>(ND/50-100/ND)	Yes<br>(below EFSA's regulations)
Trans fatty acids (g/100kcal)*	0.16<br>(ND/Max 3/ND)	No
Carbohydrates (g/100 kcal)*	13.42<br>(ND/9-14/ND)	No
Protein (g/100 kcal)*	3.65<br>(3-5.5/1.8-2.5/1.6 -5.5)	Yes<br>(exceeding EFSA's regulations)
Chlorides (mg/100 kcal)*	108.6<br>(Min 55/60-160/ Min 55)	No
Growing-up formulas		
Total fat (g/100kcal)*	3.37<br>(3-6/4.4-6/3-6)	No
Total SFA (g/100g)	73.01<br>(No regulations)	-
Palmitic acid (g/100g)	50.42<br>(No regulations)	-
Total MUFA (g/100g)	24.82<br>(No regulations)	-
Erucic Acid (g/100kcal)*	<0.1<br>(ND/Max 0.4/ND)	No
Total PUFA (g/100g)	2.67<br>(No regulations)	-

Linoleic acid (mg/100 kcal)*	6.61<br>(Min 300/500-1200/Min 300)	Yes<br>(below all regulations)
Linolenic acid (mg/100kcal)*	44.08<br>(ND/50-100/ND)	Yes<br>(below EFSA's regulations)
Trans fatty acids (g/100kcal)*	0.29<br>(ND/Max 3/ND)	No
Carbohydrates (g/100 kcal)*	14.3<br>(ND/9-14/ND)	Yes<br>(exceeding EFSA's regulations)
Protein (g/100 kcal)*	3.07<br>(3-5.5/1.8-2.5/1.6 -5.5)	Yes<br>(exceeding EFSA's regulations)
Chlorides (mg/100 kcal)*	127.83<br>(Min 55/60-160/Min 55)	No
Extra care formulas		
Total fat (g/100kcal)*	4.06<br>(4.4-6/3-6/ND)	Yes<br>(below Codex's regulations)
Total SFA(g/100g)	77.72<br>(No regulations)	-
Palmitic acid (g/100g)	32.7<br>(No regulations)	-
Total MUFA (g/100g)	21.41<br>(No regulations)	-
Erucic Acid (g/100kcal)*	0.13<br>(Max 1/ Max 0.4/ ND)	No
Total PUFA (g/100g)	2.53<br>(No regulations)	-
Linoleic acid (mg/100 kcal)*	23<br>(300 -1400/500-1200/ND)	Yes<br>(below all regulations)
Linolenic acid (mg/100kcal)*	33.45<br>(Min 50/50-100/ ND)	Yes<br>(below all regulations)
Trans fatty acids (g/100kcal)*	0.18<br>(Max 3/Max 3/ND)	No
Carbohydrates (g/100 kcal)*	13.14<br>(9-14/9-14/ND)	No

Protein (g/100 kcal)*	2.69<br>(1.8-3/1.8-2.5/ND)	Yes<br>(exceeding EFSA's regulations)
Chlorides (mg/100 kcal)*	110.83<br>(50-160/60-160/ND)	No
Milky cereal		
Total calories from fat (Calories)**	75.6<br>(83.84/ND/ ND)	Yes<br>(below Codex's regulations)
Total Fat (g/100g)	8.4<br>(ND/ND/10-25)	Yes<br>(below Libnor's regulations)
Total SFA (g/100g)	75.38<br>(No regulations)	-
Palmitic acid (g/100g)	59.96<br>(No regulations)	-
Total MUFA (g/100g)	21.4<br>(No regulations)	-
Total PUFA (g/100g)	4.06<br>(No regulations)	-
Linoleic acid (mg/100 kcal)*	69.17<br>(Min 333/ND/ Min 333)	Yes<br>(below all regulations)
Linolenic acid (mg/100kcal)*	4.77<br>(No regulations)	-
Trans fatty acids (g/100kcal)*	0.11<br>(No regulations)	-
Carbohydrates (g/100 kcal)*	16.93<br>(No regulations)	-
Calories from protein ***	59.84<br>(25.14-62.88/ND/ND)	No
Protein (g/100 kcal)	14.96<br>(ND/ND/15)	Yes<br>(below Libnor's regulations)
Chlorides (mg/100 kcal)*	100.19<br>(No regulations)	-
Cereal Meal		
Total calories from fat (Calories)**	28<br>(79.94/ND/ND)	Yes<br>(below Codex's regulations)

Total Fat (g/100g)	3.11<br>(ND/ND/10-25)	Yes<br>(below Libnor's regulations)
Total SFA (g/100g)	66.15<br>(No regulations)	-
Palmitic acid (g/100g)	48.12<br>(No regulations)	-
Total MUFA (g/100g)	21.21<br>(No regulations)	-
Total PUFA (g/100g)	4.36<br>(No regulations)	-
Linoleic acid (mg/100 kcal)*	20<br>(Min 333/ND/Min 333)	Yes<br>(below all regulations)
Linolenic acid (mg/100kcal)*	2.50<br>(No regulations)	-
Trans fatty acids (g/100kcal)*	0.34<br>(No regulations)	-
Carbohydrates (g/100 kcal)*	19.01<br>(No regulations)	-
Calories from protein (Calories)***	38.4<br>(24-56/ND/ND)	No
Protein (g/100 kcal)	9.6<br>(ND/ND/15)	No
Chlorides (mg/100 kcal)*	37.52<br>(No regulations)	No
Cornflakes		
Total calories from fat (Calories)**	21.42<br>(77.16/ND/ND)	Yes<br>(below Codex's regulations)
Total Fat (g/100g)	2.38<br>(ND/ND/10-25)	Yes<br>(below Libnor's regulations)
Total SFA (g/100g)	47.02<br>(No regulations)	-
Palmitic acid (g/100g)	25.4<br>(No regulations)	-
Total MUFA (g/100g)	24.34	-

	(No regulations)	
Total PUFA (g/100g)	9.86<br>(No regulations)	-
Linoleic acid (mg/100 kcal)*	49.24<br>(Min 333/ND/Min 333)	Yes<br>(below all regulations)
Linolenic acid (mg/100kcal)*	5.18<br>(No regulations)	-
Trans fatty acids (g/100kcal)*	0.21<br>(No regulations)	-
Carbohydrates (g/100 kcal)*	21.71<br>(No regulations)	-
Calories from protein (Calories)***	30.2<br>(23.14-57.87/ND/ND)	No
Protein (g/100 kcal)*	7.55<br>(ND/ ND/15)	Yes<br>(below Libnor's regulations)
Chlorides (mg/100 kcal)*	228.09<br>(No regulations)	-
Biscuits		
Total calories from fat (Calories)**	104.2<br>(87.45/ND/ND)	No
Total Fat (g/100g)	11.58<br>(ND/ND/10-25)	No
Total SFA (g/100g)	68.85<br>(No regulations)	-
Palmitic acid (g/100g)	50.9<br>(No regulations)	-
Total MUFA (g/100g)	27.05<br>(No regulations)	-
Total PUFA (g/100g)	3.94<br>(No regulations)	-
Linoleic acid (mg/100 kcal)*	18.29<br>(Min 333/ND/Min 333)	Yes<br>(below all regulations)
Linolenic acid (mg/100kcal)*	3.20<br>(No regulations)	-

Trans fatty acids (g/100kcal)*	0.22<br>(No regulations)	-
Carbohydrates (g/100 kcal)*	76.28<br>(No regulations)	-
Calories from protein (Calories)***	27.88<br>(26.23-65.6/ND/ND)	No
Protein (g/100 kcal)*	6.97<br>(ND/ND/15)	Yes<br>(below Libnor's regulations)
Chlorides (mg/100 kcal)*	86.90<br>(No regulations)	-
Fruit puree		
Total calories from fat (Calories)**	6.84<br>(14.56/ND/ND)	Yes<br>(below Codex's regulations)
Total Fat (g/100g)	0.76<br>(ND/ND/10-25)	Yes<br>(below Libnor's regulations)
Total SFA (g/100g)	8.78<br>(No regulations)	-
Palmitic acid (g/100g)	4.72<br>(No regulations)	-
Total MUFA (g/100g)	5.19<br>(No regulations)	-
Total PUFA (g/100g)	2.58<br>(No regulations)	-
Linoleic acid (mg/100 kcal)*	13.73<br>(Min 333/ND/Min 333)	Yes<br>(below all regulations)
Linolenic acid (mg/100kcal)*	2.74<br>(No regulations)	-
Trans fatty acids (g/100kcal)*	0.04<br>(No regulations)	-
Carbohydrates (g/100 kcal)*	15.16<br>(No regulations)	-
Calories from protein (Calories)***	5.2<br>(4.36-11/ND/ND)	No
Protein (g/100 kcal)*	1.3	Yes

	(ND/ND/15)	(below Libnor's regulations)
Chlorides (mg/100 kcal)*	411.91<br>(No regulations)	-
Vegetables and legumes puree		
Total calories from fat (Calories)**	7.65<br>(9.4/ND/ND)	Yes<br>(below Codex's regulations)
Total Fat (g/100g)	0.85<br>(ND/ND/10-25)	Yes<br>(below Libnor's regulations)
Total SFA (g/100g)	12.45<br>(No regulations)	-
Palmitic acid (g/100g)	7.76<br>(No regulations)	-
Total MUFA (g/100g)	19.01<br>(No regulations)	-
Total PUFA (g/100g)	1.53<br>(No regulations)	-
Linoleic acid (mg/100 kcal)*	8.51<br>(Min 333/ND/Min 333)	Yes<br>(below all regulations)
Linolenic acid (mg/100kcal)*	4.25<br>(No regulations)	-
Trans fatty acids (g/100kcal)*	0.33<br>(No regulations)	-
Carbohydrates (g/100 kcal)*	17.65<br>(No regulations)	-
Calories from protein (Calories)***	6.12<br>(2.82-7.05/ND/ND)	No
Protein (g/100 kcal)	1.53<br>(ND/ND/15)	Yes<br>(below Libnor's regulations)
Chlorides (mg/100 kcal)*	1021.27<br>(No regulations)	-
Meat or fish puree with vegetables		
Total calories from fat (Calories)**	19<br>(14.22/ND/ND)	No

Total Fat (g/100g)	2.1<br>(ND/ND/10-25)	Yes<br>(below Libnor's regulations)
Total SFA (g/100g)	25.35<br>(No regulations)	-
Palmitic acid (g/100g)	15.3<br>(No regulations)	-
Total MUFA (g/100g)	52.5<br>(No regulations)	-
Total PUFA (g/100g)	22.25<br>(No regulations)	-
Linoleic acid (mg/100 kcal)*	492.26<br>(Min 333/ND/Min 333)	No
Linolenic acid (mg/100kcal)*	126.58<br>(No regulations)	-
Trans fatty acids (g/100kcal)*	0.24<br>(No regulations)	-
Carbohydrates (g/100 kcal)*	13.78<br>(No regulations)	-
Calories from protein (Calories)***	12.4<br>(4.26-10.66/ND/ND)	Yes<br>(exceeding Codex's regulations)
Protein (g/100 kcal)*	3.1<br>(ND/ND/15)	Yes<br>(below Libnor's regulations)
Chlorides (mg/100 kcal)*	829.81<br>(No regulations)	-
Milk based puree		
Total calories from fat (Calories)**	23.4<br>(18.5/ND/ND)	No
Total Fat (g/100g)	2.6<br>(ND/ND/10-25)	Yes<br>(below Libnor's regulations)
Total SFA (g/100g)	70.15<br>(No regulations)	-
Palmitic acid (g/100g)	32.4<br>(No regulations)	-
Total MUFA (g/100g)	23.2	-

	(No regulations)	
Total PUFA (g/100g)	6.05<br>(No regulations)	-
Linoleic acid (mg/100 kcal)*	129.72<br>(Min 333/ND/Min 333)	Yes<br>(below all regulations)
Linolenic acid (mg/100kcal)*	10.81<br>(No regulations)	-
Trans fatty acids (g/100kcal)*	0.82<br>(No regulations)	-
Carbohydrates (g/100 kcal)*	15.24<br>(No regulations)	-
Calories from protein (Calories)***	13<br>(5.55-13.87/ND/ND)	No
Protein (g/100 kcal)*	3.25<br>(ND/ND/15)	Yes<br>(below Libnor's regulations)
Chlorides (mg/100 kcal)*	864.86<br>(No regulations)	-

Values are mean ± SD; Min: minimum; Max: maximum; C: concentration; ND: not determined

*Calculated in mg or g/100 kcal to compare with the regulations: (measured value (g/100g) / total calories (100g)) × 100

**Calculated to compare with Codex (based on fat recommendation of at least 20% of total energy).

***Calculated to compare with Codex (based on protein recommendation of 6-15% of total energy).

+ https://www.legislation.gov.uk/eur/2016/127/contents?view=plain

^ https://www.fao.org/fao-who-codexalimentarius/sh-proxy/en/?lnk=1&url=https%253A%252F%252Fworkspace.fao.org%252Fsites%252Fcodex%252FStandards%252FCXS%2B72-1981%252FCXS_072e.pdf

^ https://www.fao.org/fao-who-codexalimentarius/sh-proxy/en/?lnk=1&url=https%253A%252F%252Fworkspace.fao.org%252Fsites%252Fcodex%252FStandards%252FCXS%2B156-1987%252FCXS_156e.pdf

Table S4. Composition of infant formulas and baby food products from different countries in the world

Composition / Country	Total energy (Kcal/100g)	Total Fat (g/100g)	SFA	Palmitic acid (g/100g)	TFA	Total Carbohydrates (g/100g)	Added sugar	Protein (g/100g)	References
Eastern Mediterranean Region Data									
Egypt (2016)	ND	IF: 18.7-26.7	ND	ND	ND	IF: 64.92	ND	IF: 8.88	(Kotb, Farahat, & El-Daree, 2016)
Egypt (2014)	ND	ND	ND	ND	ND	IF: 53.04	ND	IF: 11.07	(Shenana, El-Alfy, Sania, & Gemiel, 2014)
Egypt (2006)	ND	FP+VLP: 0.15-2.18	ND	ND	ND	ND	ND	ND	(Youssef, Atwa, Bassuony, & Abol Ela, 2006)
Kuwait (2016)	ND	IF: 0.23-5.2	ND	ND	ND	ND	ND	IF:13.48	(Bu-Hamdi, Al-Harbi, & Anderson, 2016)
Lebanon	IF: 432-508<br>SF:489.5<br>FUF:460.3<br>GUF:453.7<br>ECF:478.2<br>Biscuits:437.28<br>VLP: 47<br>MFVP: 71.1<br>CM: 399.7	IF: 6.4-25.2<br>SF: 21.1<br>FUF: 16.2<br>GUF: 15.3<br>ECF: 19.46<br>FP: 0.1-2.7<br>VLP: 0.85<br>MFVP: 2.1<br>Biscuits: 11.58<br>CM: 3.11	IF: 76.29<br>SF: 74.59<br>ECF: 77.72<br>Biscuits: 68.85<br>CM: 66.15	IF: 11.4-68.2	IF: 0.38	IF: 63.18<br>MC: 70.98<br>Biscuits: 76.28<br>CM: 76<br>MFVP: 9.8<br>VLP: 8.3	Biscuits: 21.75<br>Cereal meal: 24.84	IF:13.85<br>MC:14.96<br>MFVP: 3.1<br>VLP: 1.53	Current study.
Pakistan (2021)	ND	IF: 9.82-26.63	ND	ND	ND	ND	ND	IF:12.61	(Saeed et al., 2021)
Pakistan (1985)	IF: 428-473	IF: 18.2-27	ND	ND	ND	IF:52<br>MC:74.6	ND	IF:19.65<br>MC:12.5	(Khans & Kissana, 1985)
Saudi Arabia (2022)	SF: 481.68<br>FUF: 467.26<br>GUF: 467.06	SF: 23.27<br>FUF: 20.06<br>GUF: 20.36	ND	ND	ND	IF: 55	ND	IF:15.17	(Alfaris, Alothman, Aldayel, Wabaidur, & Altamimi, 2022)
Sudan (2013)	ND	ND	ND	IF:19.99-36.92	ND	ND	ND	ND	(Bakeet, Arzoo, & Taha, 2013)
International Data									
Brazil (2017)	ND	ND	SF:42.3 ECF:41.9	SF:19.18<br>ECF:17	ND	ND	ND	ND	(Mendonça et al., 2017)
Canada (2011)	ND	ND	ND	ND	ND	ND	Biscuits: 19	ND	(Elliott, 2011)
Cote d'Ivoire (2019)	ND	ND	ND	FUF:21.7-27.79	ND	ND	ND	ND	(Kpaibé et al., 2019)
Italy (2015)	ND	IF:26.2	ND	ND	ND	IF:56.3	ND	IF:10.9	(Zunin et al., 2015).
Spain (2022)	ND	ND	ND	ND	ND	ND	CM:18.1	ND	(Garro-Mellado, Guerra-Hernández, & García-Villanova, 2022)

Spain (2019)	ND	ND	IF:37.31	IF:23.09	IF:0.03	ND	ND	ND	(Sánchez-Hernández et al., 2019)
Spain (2019)	ND	ND	IF:35.9	ND	IF:0.33	ND	ND	ND	(Martínez et al., 2019)
Turkey (2020)	Biscuits:468.3<br>CM:386	Biscuits:19.7<br>CM:4.3	ND	ND	ND	Biscuits:67.3<br>CM:76.2	Biscuits:31.4<br>CM:23.8	ND	(Güzel et al., 2020).
United Kingdom (2015)	Meat based: 61.5<br>Vegetable based: 61.2	Meat based: 2.1<br>Vegetable based: 2.5	ND	ND	ND	Meat based: 7.4<br>Vegetable based: 7.4	ND	Meat based: 3.2<br>Vegetable based:2	(Zand et al., 2015).
USA (2002)	ND	ND	IF:41.05	IF:16.2	IF:1.3	ND	ND	ND	(Satchithanandam et al., 2002).

ND: Not determined

IF: Infant formulas, SF: starting formulas, FUF: follow up formulas, ECF: extra care formulas, MC: milky cereal, PF: pureed food, FP: fruit pureed, VLP: vegetables and legumes pureed, CM: Cereal meal, MFVP: Meat or fish puree with vegetables, VLP: Vegetables and legumes puree,

[Number-number] = the range of macronutrients distribution in infant formulas and baby food products

[Number] = mean of macronutrients distribution in infant formulas and baby food products
